# Sublingual gland neoplasms: clinicopathological study of 8 cases

**DOI:** 10.4317/medoral.24634

**Published:** 2021-08-19

**Authors:** Michał Gontarz, Marta Urbańska-Gąsiorowska, Jakub Bargiel, Krzysztof Gąsiorowski, Tomasz Marecik, Paweł Szczurowski, Jan Zapała, Grażyna Wyszyńska-Pawelec

**Affiliations:** 1 Department of Cranio-Maxillofacial Surgery, Jagiellonian University Medical College, Cracow, Poland. University Hospital, Cracow, Poland; 2 Amethyst Radiotherapy Centre in Cracow, Poland

## Abstract

**Background:**

Sublingual gland neoplasms are very rare and the majority of them are malignant. The aim of this study was to evaluate the clinical course, treatment, and outcomes of these uncommon neoplasms based on the authors’ experience and the recent literature.

**Material and Methods:**

The medical charts of 8 patients with primary epithelial sublingual gland tumors treated between 1994 and 2020 were reviewed.

**Results:**

Malignant tumors comprised 75% (6/8) of cases. Adenoid cystic carcinoma was the most common (50%, 3/6) and characterized by high risk of local recurrence and lung metastasis. Pleomorphic adenoma was the only representative of benign tumors with no evidence of local recurrence in follow up.

**Conclusions:**

Treatment of choice of sublingual gland tumors is surgery. However, due to the fact that adenoid cystic carcinoma is the most common malignancy with poor prognosis, surgical treatment should be combined with postoperative radiotherapy. Benign sublingual tumors are less common and treatment of choice in these cases is tumor resection together with sublingual gland.

** Key words:**Salivary gland neoplasms, sublingual gland, pleomorphic adenoma, adenoid cystic carcinoma, mucoepidermoid carcinoma.

## Introduction

The sublingual glands are the smallest of the major salivary glands. The most common pathology of the sublingual glands is ranula comprising an extravasation of saliva from the sublingual gland due to trauma of Rivinus duct. Sublingual gland neoplasms are very rare and comprise about 1% of all epithelial salivary gland tumors (1). Unfortunately, most of them are malignant and adenoid cystic carcinoma (ACC) is the most common histological type followed by mucoepidermoid carcinoma (MEC) (1-3). Other histological types such as acinic cell carcinoma, carcinoma ex pleomorphic adenoma, polymorphous low grade adenocarcinoma, primary squamous cell carcinoma, salivary duct carcinoma, clear cell carcinoma, basal cell carcinoma, mucinous adenocarcinoma, papillary cystadenocarcinoma and NUT carcinoma are extremely rare (1,4-7). Benign tumors are usually represented by pleomorphic adenoma (PA) followed by basal cell adenoma (7).

This study presents case series of epithelial sublingual gland tumors in patients operated in 26-year period. Additionally, a review of the recent literature was conducted in order to evaluate epidemiological data of sublingual gland tumors.

## Material and Methods

Between January 1994 and December 2020, 822 patients were treated for primary epithelial salivary gland tumors in the Department of Cranio-Maxillofacial Surgery of the Jagiellonian University in Cracow. The sublingual glands tumors were observed in 8 cases (0.97%). The medical charts of the patients were evaluated according to demographic characteristics, clinical presentation, histopathological aspects, methods of treatment, recurrences, follow-up, and outcomes. This study was approved by the institutional review board (No. 122.6120.287.2016). Because only medical files were obtained, the review board approved the study without the need for patient consent as long as all personal information was kept confidential. In addition to the review of medical charts, the English language literature published from January 2000 to December 2020 was reviewed to identify epidemiological data for these uncommon tumors.

## Results

**Figure 1:**
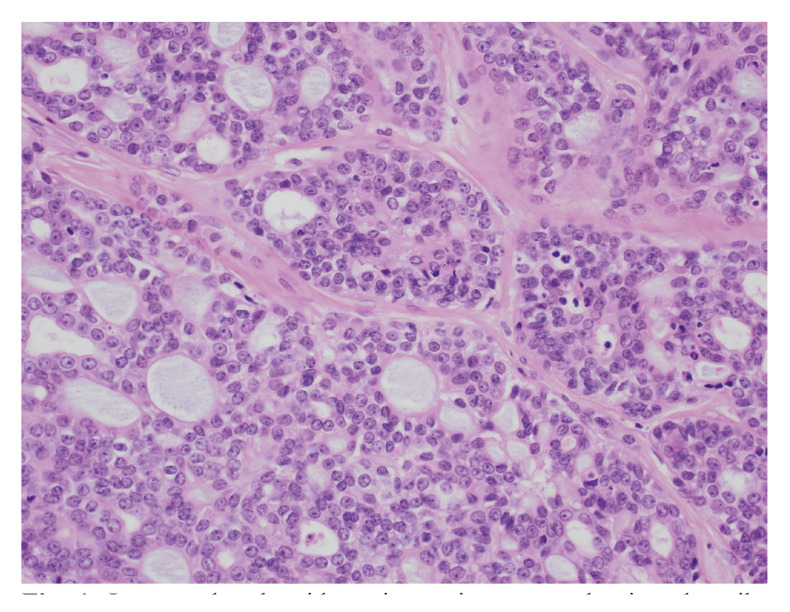
Low grade adenoid cystic carcinoma, predominantly cribriform, with myxoid material in the pseudoglandular spaces. H&E stained. Magn. 880x.

The group comprised 4 female patients and 4 male patients. The age of the patients ranged from 14 to 64 years, with an average of 43.1 years (median 46.5). Malignant tumors comprised 75% of cases (Fig. 1). All of the patients were of white ethnicity. Patient’s characteristics, treatment and follow up are presented in Table 1.

**Table 1:**
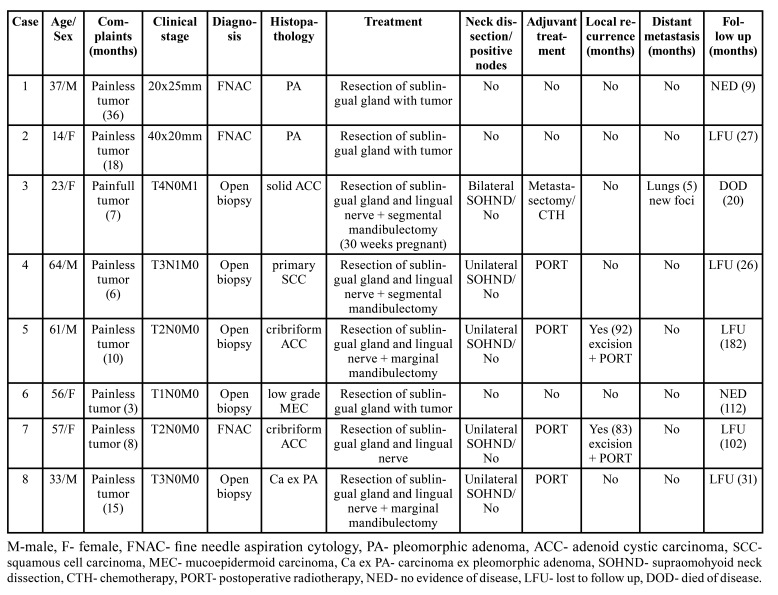
Clinical characteristic of patients with sublingual gland tumors.

## Discussion

Sublingual gland neoplasms are rare. A few large series showing histological type of these tumors have been published in the recent literature (Table 2) (2-4,7-15). In some epidemiological studies authors did not observed sublingual tumors at all. Young profile of patient was characteristic in our cohort (mean age 43.1 years). The youngest patient was a 14 year old girl with PA, other 23-year old female with ACC was operated in the 30th week of pregnancy. However, in most studies mean age of patents is around 50-55 years (2,3,15,16).

**Table 2:**
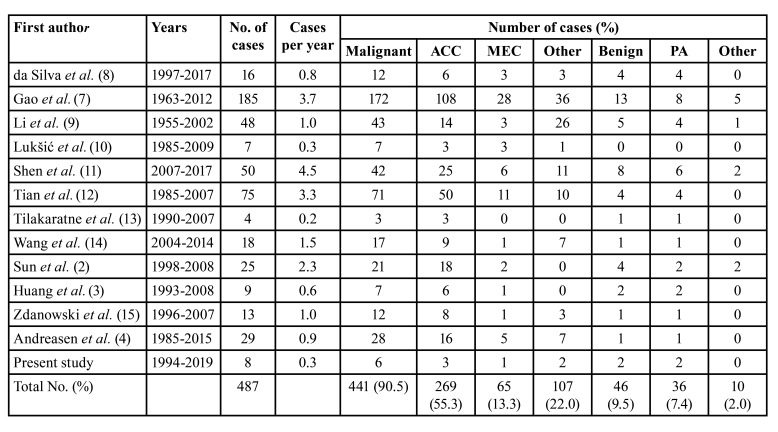
Distribution of sublingual gland tumors: review of the literature.

In contrast to the parotid and submandibular glands, the percentage of malignant sublingual gland tumors is much higher, ranging from 75% to 100% in the literature. In our study, neoplasms of the sublingual gland accounted for approximately 1% (8/822) of cases, of which 75% (6/8) were malignant. In accordance with the literature review, the most common malignant neoplasm in the sublingual gland is an ACC. Based on our analysis ACC comprises 55.3% (269/487) of all and 61.1% (269/441) of malignant tumors (Table 2). Most of them are cribriform ACC followed by tubular and solid ACC (3,15,16). MEC is the second most common malignancy. In contrast to other sites high grade MEC is observed more common in sublingual gland (2,3,16). Based on the literature almost all histological malignancies can be observed in sublingual gland. However, they are extremely rare. On the other hand, in this cohort the only histologically benign tumor in the sublingual gland was a PA, the same as in other studies (3,4,15,16). In accordance with the literature PA comprises 7.4% (36/487) of all and 78.2% (36/46) of benign tumors (Table 2). Unusual benign tumors include: basal cell adenoma, myoepithelioma, cystadenoma and oncocytoma (2,7,9,11).

**Figure 2:**
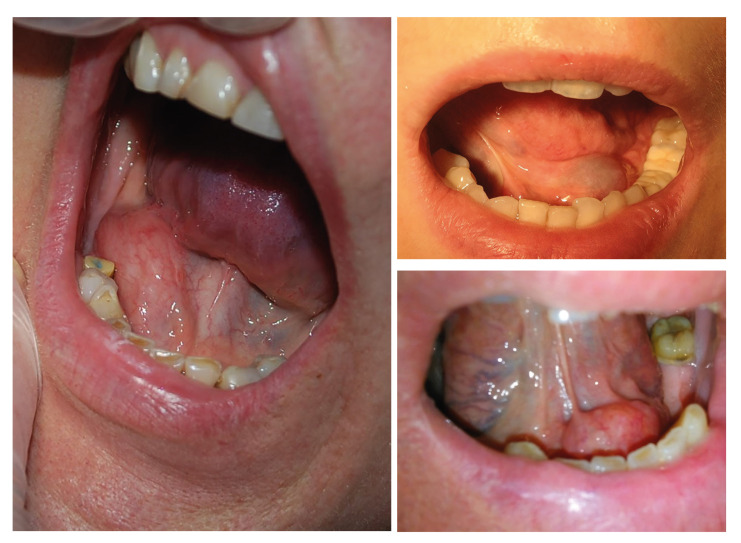
Clinical pathology ​​of the sublingual gland. a) Adenoid cystic carcinoma; b) Oral (simple) ranula; c) Sialolithiasis.

Sublingual gland tumors are usually asymptomatic and cause a painless swelling in the floor of the mouth. They can be detected accidentally during a dental examination. Other symptoms reported by the patient include pain, problems with dental prosthesis retention and tongue numbness (1,17). Often, thorough clinical examination allows to determine the nature of the pathology in the area of ​​the sublingual gland (Fig. 2).

Computed tomography (CT) or magnetic resonance (MR) imaging allows the assessment of the solid or cystic nature of the tumor and possible infiltration of the mandibular body and eventual cervical metastases in case of malignancy. Histological diagnosis is most often established on the basis of fine-needle cytology (FNAC), characterized by 69.2% of sensitivity for malignancy or open biopsy (4). Surgical treatment is the treatment of choice for sublingual gland tumors. Benign neoplasms are excised with the entire sublingual gland by intraoral approach in order to reduce the risk of local recurrence and ranula development. Sublingual low grade cancers can be treated by local excision with the gland without elective neck dissection in clinically negative neck (cN0). High grade cancers with cN0 require elective neck dissection in III to I cervical levels due to the risk of occult metastasis. In clinically positive neck (cN+) comprehensive radical neck dissection should be consider (1,2). According to Spiro (17) tumors above 2 cm of diameter require pull-through, *en bloc* resection. Also due to the high incidence of ACC in sublingual gland and risk of perineural invasion lingual nerve should be resected with frozen section examination of the proximal nerve stump. In addition, in case of infiltration of periosteum, marginal mandibulectomy is sufficient but evident bone invasion requires segmental mandibulectomy. Defect reconstruction depends on its size. Small soft tissue defects can be easily closed by transmandibular suturing or local flaps (facial artery musculomucosal flap- FAMM, deep lingual artery axial propeller flap) (17-19). Large soft tissue defects with marginal mandibulectomy can be reconstructed by free radial forearm flap (FRFF) (2). In case of mandibulectomy with wide soft tissue excision the reconstruction of choice are free flaps (deep circumflex iliac artery- DCIA bone flap with internal oblique muscle or free fibular flap with skin island- FFF).

Benign salivary gland tumors have excellent prognosis after complete surgical resection and there is no role for adjuvant radiotherapy. In early stage of low grade cancer as adenocarcinoma, mucoepidermoid carcinoma, acinic cell carcinoma postoperative radiotherapy (PORT) is not indicated if adequate margins are achieved (20-28). Patients with high-risk factors such as: high-grade, advanced-stage (T3 and more) lesions, positive surgical margins, perineural, vascular or lymphatic invasion, lymph node involvement especially extracapsular extension (ECE+), skin and nerve invasion and nearly all adenoid cystic carcinomas, benefit from PORT (20-30). PORT should be started before 6 weeks after surgery. Patients with high risk of locoregional recurrence with adverse features require 60-66 Gy in 30-33 fractions during 6-6.5 weeks. Low risk patients can be treated by 50-55 Gy (20). The radiation treatment volume encompasses the tumor bed and regional lymph nodes only if involved or thought to be at high risk. For adenoid cystic carcinoma, neural pathways to the skull base are also included. Retrospective data do not provide evidence that adjuvant chemoradiotherapy is more effective than adjuvant radiotherapy (RT) alone. An analysis of over 2000 patients from the National Cancer Database failed to demonstrate any improvement in overall survival with the addition of chemotherapy to RT compared with RT alone (30). Definitive RT is reserved for patients who are medically inoperable or who have unresecTable disease (27). Although there are no randomized clinical trials, observational data indicate that adjuvant RT improves both local control and survival for selected patients (28).

In patients with ACC in current study we did not observe nodal metastasis in surgical specimen, similarly to Zdanowski *et al*. (15) research. However, positive lymph nodes in ACC in surgical specimen in different studies range from 11.1% to 50% (2-4). Long term follow up in patients with ACC is needed. In this study local recurrence of ACC was observed in long time period in two patients, 7 and 8 years after primary treatment. In accordance with the literature local recurrence of ACC is observed up to 35.3% of cases (2-4,15,16). Lung metastases are one of the main reasons of death in patients with ACC and are found in up to 33.3% of patients (3,4,15,16). For that reason, patients with ACC require CT of the chest at least once a year. In our material 23-year old female with ACC was operated in the 30th week of pregnancy with single lung metastasis. After childbirth, the patient had thoracotomy with metastasectomy followed by chemotherapy. Unfortunately, the patient developed new foci of lung metastases and died 20 months after surgery.

In conclusion, sublingual gland neoplasms are very rare, and in the vast majority are malignant. The peak of incidence is between 40 and 60 years. However, these tumors can be observed also in children and adolescents, without sex predilection. The most common histological type is ACC which requires surgical treatment combined with PORT. Benign sublingual tumors are less common and the treatment of choice in these cases is tumor resection together with sublingual gland.
